# Characterization of cocoa production, income diversification and shade tree management along a climate gradient in Ghana

**DOI:** 10.1371/journal.pone.0195777

**Published:** 2018-04-16

**Authors:** Issaka Abdulai, Laurence Jassogne, Sophie Graefe, Richard Asare, Piet Van Asten, Peter Läderach, Philippe Vaast

**Affiliations:** 1 University of Göttingen, Tropical Plant Production and Agricultural Systems Modelling (TROPAGS), Göttingen, Germany; 2 International Institute of Tropical Agriculture (IITA), Kampala, Uganda; 3 Tropical Silviculture and Forest Ecology, University of Goettingen, Büsgenweg 1, Göttingen, Germany; 4 International Institute of Tropical Agriculture (IITA), Accra, Ghana; 5 International Center for Tropical Agriculture (CIAT), Hanoi, Vietnam; 6 UMR Eco&Sols, Centre de Coopération Internationale en Recherche Agronomique pour le Développement (CIRAD), Université de Montpellier, Montpellier, France; 7 World Agroforestry Centre (ICRAF), Hanoi, Vietnam; University of Leeds, UNITED KINGDOM

## Abstract

Reduced climatic suitability due to climate change in cocoa growing regions of Ghana is expected in the coming decades. This threatens farmers’ livelihood and the cocoa sector. Climate change adaptation requires an improved understanding of existing cocoa production systems and farmers’ coping strategies. This study characterized current cocoa production, income diversification and shade tree management along a climate gradient within the cocoa belt of Ghana. The objectives were to 1) compare existing production and income diversification between dry, mid and wet climatic regions, and 2) identify shade trees in cocoa agroforestry systems and their distribution along the climatic gradient. Our results showed that current mean cocoa yield level of 288kg ha^-1^yr^-1^ in the dry region was significantly lower than in the mid and wet regions with mean yields of 712 and 849 kg ha^-1^ yr^-1^, respectively. In the dry region, farmers diversified their income sources with non-cocoa crops and off-farm activities while farmers at the mid and wet regions mainly depended on cocoa (over 80% of annual income). Two shade systems classified as medium and low shade cocoa agroforestry systems were identified across the studied regions. The medium shade system was more abundant in the dry region and associated to adaptation to marginal climatic conditions. The low shade system showed significantly higher yield in the wet region but no difference was observed between the mid and dry regions. This study highlights the need for optimum shade level recommendation to be climatic region specific.

## Introduction

Ghana is the second largest global cocoa producer with an estimated US$ 2 billion generated by export revenues in 2013 [1]. Cocoa is the main agriculture export product and sustains the livelihood of more than 800,000 small-scale households across the cocoa growing region of the country [2,3]. Over two million cocoa farmers across the West African sub-region are vulnerable to climate change [4]. Drought is traditionally one of the key climate change effects expected to negatively affect agricultural production [5]. The cocoa landscape in Ghana has experienced a latitudinal shift since early 1980s with more than half of current national cocoa production coming from the climatically more suitable areas of the Southwestern region that harbour remnants of the rainforest [2,6]. The relatively marginal areas of Northern Ashanti and Brong Ahafo regions used to exhibit good climate suitability and produced more cocoa than the Western region prior to the extreme climate events in 1983/4 that resulted in severe drought and wildfires [2,7]. Cote d’Ivoire, the world largest cocoa producer, has also experienced a similar shift with such extreme climate event being a contributing factor to geographic shifts in production areas [8]. Such spatial and temporal variations in climatic events allow for the identification of farmers responses to climate change [4,8,9]. An increase in dry season maximum temperatures together with seasonal droughts are the main projected impacts of climate change within the West African cocoa belt [4,10,11]. The variability in current climatic conditions within the cocoa belt of Ghana and Cote d’Ivoire has resulted in different levels of climate suitability [10]. Different types of climate change adaptation including incremental, systemic and transformative adaptation would be required at different locations within the cocoa belt of West Africa depending on the projected climatic changes [9]. Good to very good cocoa climate suitability is projected to continue shifting cocoa production to the more wet forest regions by 2050 [4,9,10]. A lack of available forest land for new cocoa farms in the wet regions and the potentially negative effects of climate change are foreseen as being part of the major constraints to sustainable production growth in the coming years [12,13].

There is a need to increase the adoption of climate smart technologies in cocoa to sustain its production [6,14]. Understanding the existing characteristics of cocoa production, perceived climate change and drought effects, income diversification and management of shade trees in cocoa growing systems in different climatic regions within the cocoa belt should be the first step toward the design of promising adaptation pathways [15]. Knowledge of existing cocoa agroforestry systems is important in developing interventions and tools to aid farmers with proper management strategies. A key determinant of farming systems is the variation of agro-ecological zones in which the crops are cultivated [16] and hence characterizing cocoa production systems along a climatic gradient becomes justified. Characterization of shade tree species in cocoa growing systems is an important component of the identification of existing systems [17]. This could support location and system specific adaptation since agro-ecological zones for cocoa are being altered by changing climatic conditions, especially in areas close to the forest-savannah transition zones [4]. In the context of climate change, shaded cocoa systems have been identified as an appropriate strategy for increasing resilience and improving agro-ecosystem functioning at plant, plot and landscape levels [10,12,13,18–21]. Although potential soil water competition between shade and cocoa trees in cocoa agroforestry systems have been noted as a potential limitation under marginal cocoa climate [22]. Lower yields have also been reported for agroforestry systems under relatively lower rainfall locations in the Ashanti region of Ghana [23,24], this could be compensated via income diversification. Consequently, the documentation of agroforestry systems distribution between agro-ecological zones is a necessary step towards identifying and optimizing climate change adaptation strategies.

The aim of this study was to characterize cocoa production, perceived climate change effect on cocoa production, income diversification and shade tree management in cocoa growing systems along a climate gradient in Ghana. The studied locations represented areas with projected future (2050) climatic suitability of medium to high, low to medium and low to non-suitable from the wet to the dry region respectively [10]. The first objective was to compare the regions in terms of (a) current cocoa production, (b) farmers’ perception on the effects of climate change and drought on cocoa production, and (c) cocoa farmers’ income diversification. Ultimately, our second objective was (a) to document the presence of shade tree species and their functions and (b) to characterize the existing cocoa agroforestry systems and their distribution along the climatic gradient. These objectives were based on the assumptions that location along a climate gradient greatly influences cocoa production, cocoa farmer income diversification and type of shade tree management by farmers.

## Materials and methods

### Ethics

Our study conformed to the current laws of Ghana and international research rules. The appropriateness of the study methodology was reviewed and approved by the ethics committee of the International Institute for Tropical Agriculture (IITA). Permission was asked from all participating farmers before the commencement of interviews, on farm surveys and sampling. All selected farmers verbally consented to participate.

### Study area

**Fig 1:**
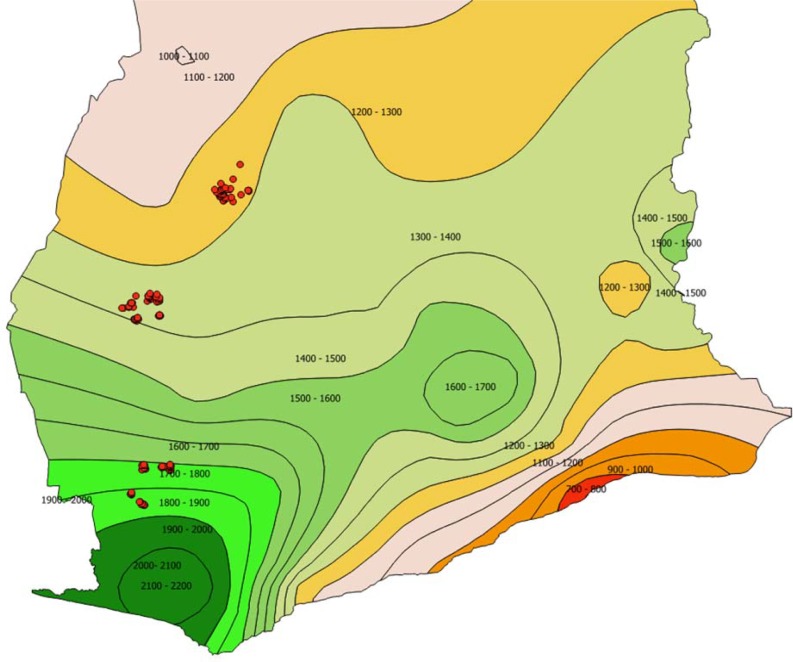
Mean annual rainfall distribution (mm) across Southern Ghana with GPS points of studied sites marked with red dots, representing the dry, mid and wet regions from North to South. Rainfall distribution map of Ghana accessed from [30]. Available in public domain.

The study was conducted in three regions within the cocoa belt of Ghana along a climatic gradient represented as dry, mid and wet (Fig 1). The dry region was Akumadan and Afrancho communities in the Offinso North district of the Ashanti region. The vegetation here used to be semi-deciduous but has changed to a forest savanna due to frequent fires and extensive agricultural practices [25]. The region has an extended dry period from November to March, during which wildfires are common and occasionally destroy farms [26]. This region is well known for tomato production, and recently also for cassava and maize [25]. Cashew is a developing tree crop in this region, and may potentially replace cocoa due to increasing pronounced savanna conditions in this region. The mid region comprised selected communities around Goaso in the Asunafo North district of the Brong Ahafo region. The wet region consisted of communities surrounding Asankragua in the Wassa Amenfi West district of the Western region (Fig 1). The mid and wet regions are within the moist semi-deciduous and the moist evergreen vegetation types [27]. In these regions, the dry season lasts from November to February and relative humidity increases towards the south [2].

The gradient is characterized by annual rainfall of 700–1200 mm in the dry region, 1250–1750 mm in the mid region, and 1400–2000 mm in the wet region [25,28,29]. Mean temperature variation is less pronounced, with 27, 25 and 26°C from dry to wet respectively.

### Sampling and data collection

Data collection took place from April to September 2014 with fifty (50) cocoa farmers selected per region for interviews and on-farm inventories. Farmers from the dry and mid regions were randomly selected from a database of the Kuapa Kokoo Farmers’union, which has about 100,000 farmers across Ghana selling Fair Trade certified cocoa. The union combines two administrative districts of Offinso North and South as one cocoa purchasing district referred to as Offinso. A total of 1814 farmers were listed in the Offinso database, but only 261 belonging to Akumadan and Afrancho in the northernmost part were selected for our study. For the mid region, the database consisted of 954 farmers out of which fifty (50) were randomly selected. For the wet region, 50 farmers were randomly selected from 1800 farmers listed in the Rainforest Alliance database managed by Agro-Eco Louis Bolk Institute (LBI).

Farmers were interviewed about household characteristics, perceived climate change and drought effects on cocoa production and income diversification strategies by using a digitized questionnaire on smart phones, which allowed data to be stored in an online database. The questionnaire covered topics such as farmer age, household size, and number of cocoa farms owned by farmers, cocoa farm size, source of planting material and cocoa farm age for both mature and young cocoa farms i.e. cocoa farms above and below 5 years respectively. From the survey, the number of farmers with young cocoa farms were 39, 26 and 35 for the dry, mid and wet regions, respectively, and hence a total of 100 respondents. Information on cocoa farm land use history was obtained to help understand changes in cocoa land availability. Farmers were further asked about quantity of dry cocoa beans (yield) produced in the 2012/13 cocoa season, which was cross-checked with their sales record books. Drought related production constraints, income distribution between cocoa, non-cocoa crops and off-farm income activities were also documented. Cocoa farm management activities such as labor cost, fertilizer, fungicide and pesticide usage were also noted.

On-farm inventories were carried out on mature cocoa farms of each farmer. In instances where a farmer had more than one mature farm, one farm (of interest) was randomly selected for inventory. Data was collected on the number of shade trees, species identity, diameter at breast height (DBH), shade tree canopy cover and mode of regeneration through a complete inventory of the entire farm. DBH was measured with a diameter tape, and canopy area was calculated by measuring the longest and shortest canopy lengths with a 30m tape. The canopy cover estimation was later validated with shade tree canopy and DBH relationships established by Asare and Ræbild [31]. Farm size was measured with Garmin GPS equipment. Cocoa tree density, and pest and disease incidence were measured at the cocoa farm center on a fixed area transects of 40 m with cocoa trees selected within 10 m perpendicular distance and at 10m interval as proposed by Nath et al. [32]. Incidence of black pod (*Phytophthora palmivora and P*. *megakarya*) and capsids (*Sahlbergella singularis* and *Distantiella theobroma)* was assessed on the cocoa trees within the established transect. Total number of both mature and immature (cherrels) cocoa pods were counted on the cocoa trees within the sampled transect. Black pod and capsid infected pods were harvested and counted. The black pod and capsid incidence per farm were then expressed as the percentage of infected pods to total pods counted per tree.

### Data analysis

Statistical analysis to assess variation in cocoa production along the climatic gradient was performed with Statistica software. Descriptive statistics and ANOVA were applied to analyze farm characteristics between regions. This was followed by a k means cluster analysis to identify clusters representing the various cocoa agroforestry systems. Statistical differences with respect to yield, shade parameters, management, pest and diseases were compared between the identified agroforestry systems in each region. Box plots were used to show the differences between the systems across regions. For identification of cocoa agroforestry systems through cluster analysis, a total of 142 of the 150 inventoried cocoa farms (due to removal of 8 outliers) were used with 45, 49 and 48 in the dry, mid and wet locations, respectively. Out of the150 farmers, 39, 26 and 35 also had young cocoa farms (< 5 years) for the dry, mid and wet regions, respectively.

## Results

### Cocoa production, income diversification and perceived negative effects of climate change and drought on cocoa production

For the 2012/13 crop season, yield in the dry region was significantly lower than the mid and wet regions with mean cocoa yields of 288, 712 and 849 kg ha^-1^ yr^-1^, respectively. The age of cocoa farmers decreased from dry to mid and wet regions, with significant difference between the dry and wet regions. This trend was positively correlated with household size, as old farmers had significantly larger family sizes. Differences in cocoa farm age were surprisingly not pronounced across the regions, but farm size was significantly larger in the mid region.

**Fig 2:**
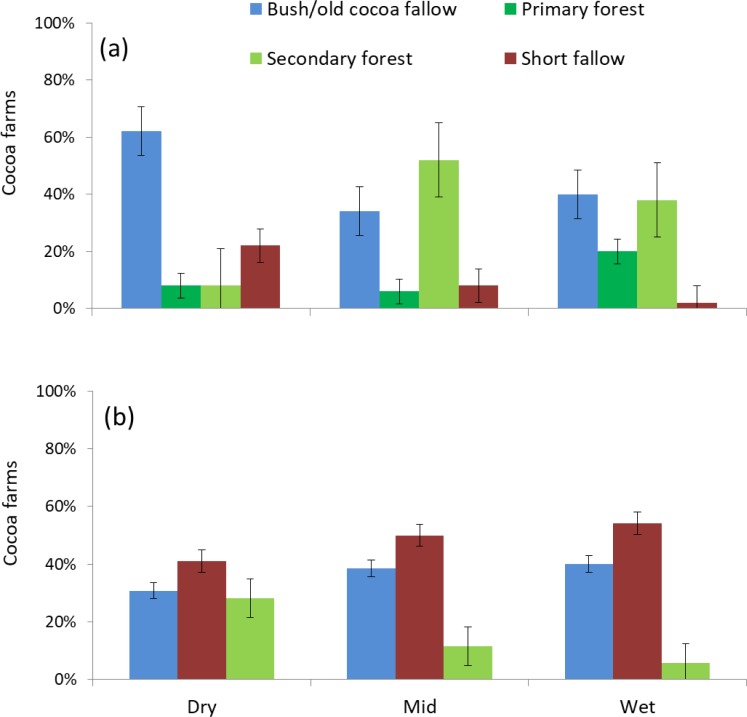
Land use history of (a) mature and (b) young (less than 5 year old) cocoa farms along a climatic gradient from dry to mid and wet regions of Ghana. N = 50 per region for mature farms. For young farms, N = 39, 26 and 35 for dry, mid and wet regions respectively.

The regions were differentiated by the cocoa farm land use history (Fig 2) with the dry region dominated by farms having been previously used for cocoa, but with a short fallow combined with annual crops such as maize and vegetables in-between cocoa cycles. The mid and wet regions had common characteristics, having been converted from primary and secondary forests. Expectedly, the highest proportion of mature cocoa farms established after deforestation of primary forest was noted in the wet region where the most recent cocoa expansion has occurred.

The size of young cocoa farms (< 5 years) was significantly smaller in the wet than the mid and dry regions. The differences in size between young and mature farms per region were not significant. None of the young cocoa farms (Fig 2B) were established on primary forest as all remaining forests are under protection. Planting material was in the form of cocoa seedlings or pods, with the source being either hybrid from the Seed Production Unit (SPU) of the Ghana Cocoa Board (COCOBOD), or from farmers’ own selections. The use of the SPU seedlings was less widespread in the wet region (Table 1).

**Table 1:** Characteristics of cocoa production along a climatic gradient from dry to mid and wet regions in Ghana (superscript letters denote significant differences between regions at p≤0.05) and no letters for variables without difference between the regions.

Location	Dry			Mid			Wet		
**Mature farms**	Mean±SD	Min.	Max.	Mean±SD.	Min.	Max.	Mean±SD	Min.	Max.
Farmer age (yrs)	64^a^ ±19	32	100	54^ab^± 14	26	90	47^bc^±11	25	71
Household members	11^a^ ±8	1.0	42	7^b^±3	1	21	8^b^±3	1	16
Number of cocoa farms per farmer	2 ±0.4	1.0	3.0	2±0.9	1.0	5.0	3±1.3	1.0	7.0
Cocoa farm size (ha)	1.7^a^ ± 2.2	0.4	16	5.7^b^±7	0.8	40	2.16^a^±1.7	0.3	8.0
Yield (ha^-1^ yr^-1^)	282^b^ ±232	14	975	712^a^± 303	177	1387	849^a^±327	338	1484
Cocoa farm age (yrs)	17 ±11	6	50	21±8	5	40	16.4±8.9	5	48
**Young cocoa farms**									
Farmer age (yrs)	64^a^ ±21	32	100	53^ab^±12	27	80	46^bc^±10	25	67
Farm size (ha)	2.1^a^ ± 2.8	0.2	16.0	2.3^a^±2.5	0.4	12.0	1.0^b^±0.7	0.1	3.6
Source of planting material									
Hybrid seedlings (%)	70±40	0.0	100	50±50	0.0	100	40±50	0.0	100
Farmer selected seedlings (%)	30 ±40	0.0	100	50±50	0.0	100	60±50	0.0	100

**Fig 3:**
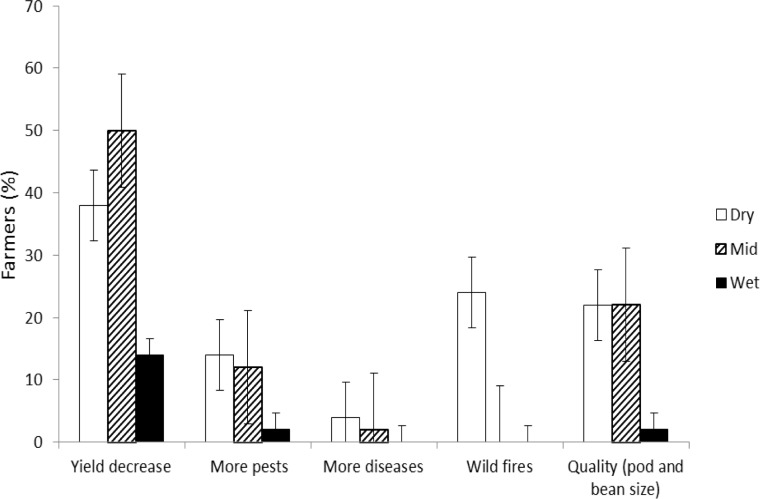
Farmers’ (%) perceived effect of drought on cocoa production from dry, mid and wet regions of Ghana.

Farmer’s perception of drought effect on cocoa production varied across regions. The perceived effects were a yield decrease, an increase in pests and diseases incidence and decrease in pod and bean quality with fire being an additional burden in the dry region (Fig 3). A yield decrease was the most often perceived effect of drought on cocoa production in all regions, but it was more often stated by farmers as such in the dry and mid regions compared to the wet region. Pests and diseases followed a similar trend but on a much lower scale. A decrease of cocoa quality was also perceived by about 25% of the farmers in the dry and mid regions.

**Fig 4:**
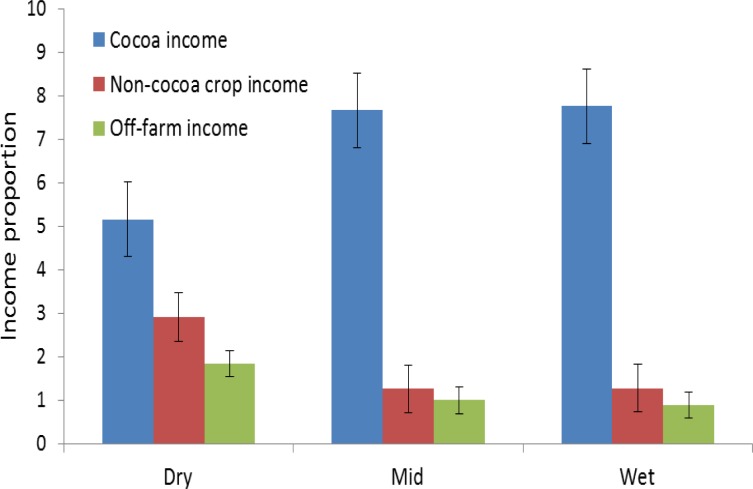
Distribution of cocoa and non-cocoa income proportions among farmers along a climate gradient from dry, mid and wet regions in Ghana.

Cocoa farmers in the dry region derived their annual income from 50% cocoa sales, 30% non-cocoa crops sales and 20% through off-farm income activities such as trading (Fig 4). Farmers in the mid and wet regions derived approximately 80% of their annual income from cocoa and 10% each for non-cocoa crops and off-farm income activities (Fig 4).

### Characterization of existing shade trees and their functions along the climatic gradient

Shade tree density, diversity and percentage shade cover were significantly higher in the dry than in the mid and wet regions (Table 2). The most common shade tree species in the dry and mid regions was avocado (*Persea Americana*) fruit tree, whereas a timber species, *Terminalia superba*, was the most common shade tree species in the wet region (Table 2). In the dry region, apart from *Antiaris toxicaria*, other most common species were two fruit trees, i.e. *P*. *americana* and *Citrus sinensis*. *Newbouldia laevis*, mainly used as live stakes for yam (*Dioscorea* spp), was the second most abundant species in the mid and wet regions (Table 2). The Cocoa Research Institute of Ghana (CRIG) has listed eight desirable and ten undesirable shade tree species for cocoa cultivation in Ghana [33]. Among the top fifteen most abundant species in the dry and mid region, only two were among those desirable species, i.e. *T*. *superba* and *Albizia ferruginea* in the dry region, and *T*. *superba and Milicia excelsa* in the mid region. In the wet region, six desirable and two undesirable CRIG recommended species, were among the top fifteen species. The desirable species included *Alstonia boonei*, *M*. *excelsa*, *Terminalia ivorensis*, *Entandrophragma angolense*, *Pycnanthus angolensis* and *T*. *superba*, whereas *Musanga cecropioides* and *Ceiba pentandra* were undesirable species. The remaining species in the dry and mid regions were neither among the desirable nor undesirable species (Table 2). The percentage of planted shade trees on farmers’ fields decreased from the dry to the wet region (Table 2), but the overall share of planted trees was low, indicating that farmers rely mainly on assisted natural regeneration for maintaining shade trees in cocoa farms.

**Table 2:** Occurence (count in 50 farms) of the fifteen most dominant shade tree species and shade characteristics along a climatic gradient from dry to mid and wet cocoa regions in Ghana. N = 50 farms per region.

Dry		No.	Mid	No.	Wet	No.
1. *Persea Americana*	166		*Persea Americana*	143	Terminalia superba *	229
2. *Antiaris toxicaria*	159		*Newbouldia laevis*	134	*Newbouldia laevis*	159
3. *Citrus senensis*	156		*Ficus capensis*	122	Milicia excelsa *	104
4. *Albizia zygia*	100		Terminalia superba *	103	*Morinda lucida*	78
5. Terminalia superba *	99		*Morinda lucida*	99	*Cola nitida*	67
6. *Morinda lucida*	84		*Sterculia tragacantha*	78	*Persea Americana*	54
7. *Holarrhena floribunda*	79		*Ficus exasperate*	68	Terminalia ivorensis *	59
8. *Sterculia tragacantha*	75		*Holarrhena floribunda*	66	Ceiba pentandra **	44
9. *Ficus exasperate*	72		Milicia excelsa *	62	Musanga cecropioides**	44
10. *Gliricidia sepium*	62		*Citrus sinensis*	61	*Ficus exasperata*	32
11. *Ficus capensis*	51		*Albizia zygia*	49	Entandrophragma angolense *	30
12. *Newbouldia laevis*	45		*Antiaris toxicaria*	41	*Rauvolfia vomitoria*	30
13. *Ricinodendron heudelotii*	41		*Ricinodendron heudelotii*	36	*Hannoa klaineana*	28
14. *Cola nitida*	39		*Amphimas pterocarpoides*	36	Pycnanthus angolensis *	28
15. *Albizia ferruginea* *	35		*Bombax buonopozense*	33	Alstonia boonei *	24
Shade tree density ha^-1^	49c		Shade tree density ha^-1^	23a	Shade tree density ha^-1^	34ab
Species ha^-1^	22c		Species ha^-1^	11a	Species ha^-1^	16ab
Shade cover (%)	27c		Shade cover (%)	13a	Shade cover (%)	18ab
Planted shade trees (%)	27c		Planted shade trees (%)	13b	Planted shade trees (%)	9ab

CRIG recommended desirable* and

undesirable** shade tree species. Different letters significantly different

**Fig 5:**
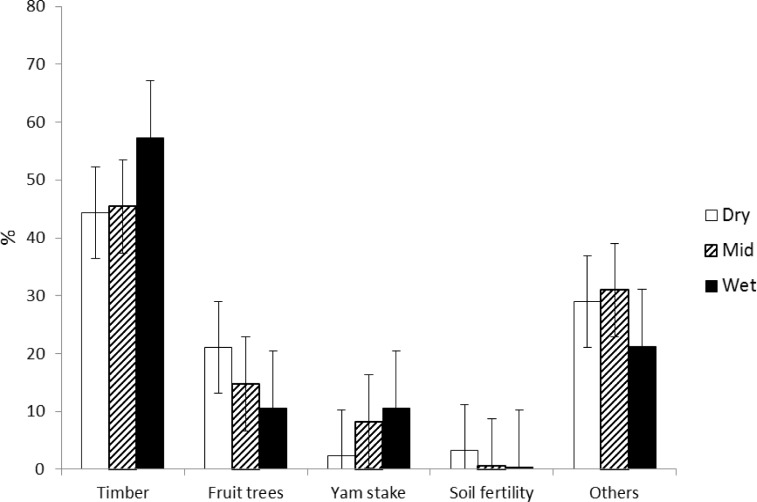
Eco-systemic functions of shade trees along a climatic gradient from dry to mid and wet cocoa regions in Ghana.

Primary functions of shade trees varied across regions (Fig 5). Number of fruit tree species decreased from the dry to the wet regions. The use of soil fertility improving shade tree species such as *Gliricidia sepium* was also mainly observed in the dry region. Use of timber and yam stake tree species was quite common, but more predominant in the wet region. Other functions include fuel wood, medicinal value, fodder and building material.

**Fig 6:**
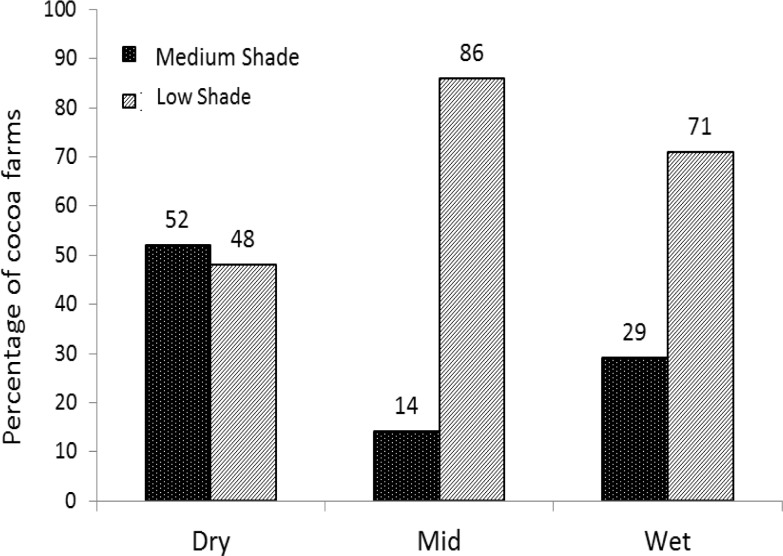
Distribution of medium and low shade cocoa systems along a climatic gradient from dry to mid and wet cocoa regions in Ghana.

**Fig 7:**
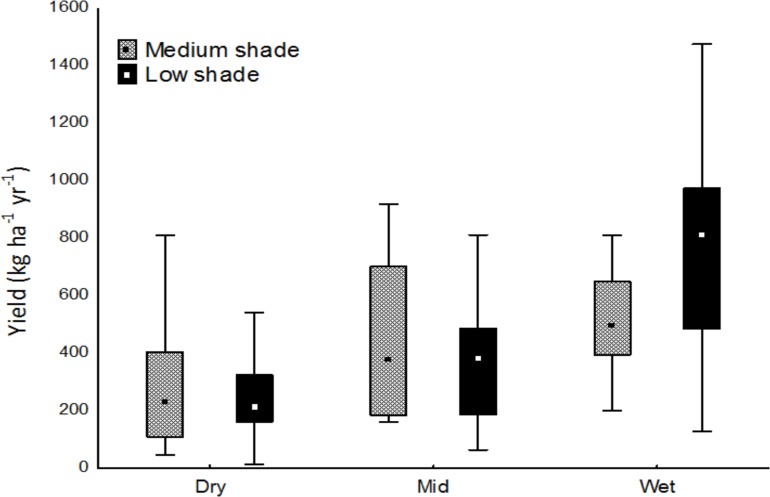
Yield variation between medium and low shade systems along a climatic gradient from dry to mid and wet cocoa regions in Ghana (different letters indicate significant differences at between systems p<0.05).

Two types of cocoa agroforestry systems were identified and referred to as medium and low shade cocoa agroforestry systems. Medium shade systems predominated in the dry region, while low shade systems were more predominant in the mid and wet regions (Fig 6). The two systems were significantly different in terms of percentage shade tree canopy cover, shade tree density and species diversity (Table 3). The medium shade system was characterized by a mean shade cover of 31%, an average diversity of 24 shade tree species ha^-1^, a density of 56 trees ha^-1^ and basal area of 6.5 m^2^ ha^-1^. The low shade system with lower values of 11% mean shade tree canopy cover, diversity of 12 shade tree species ha^-1^, density of 21 trees ha^-1^ and basal area of 3.4 m^2^ ha^-1^. There was no significant difference in DBH of the shade trees between systems for all the regions. The low shade system recorded significantly higher cocoa yield than the medium shade system in the wet region. No significant cocoa yield difference was observed between systems in the dry and mid regions (Fig 7). Timber value of shade trees could not be estimated as farmers do not have the right to sell naturally occurring timber trees on their farms. Extra income from mature cocoa farms mainly came from sales of avocado *(P*. *Americana)*, orange *(C*. *sinensis)*, banana and plantain (*Musa* spp) and yam (*Dioscorea* spp). These crops were most common in the low shade system while the medium shade system was dominated by quality timber tree species such as *T*. *superba*, *T*. *ivorensis*, *M*. *excelsa*, *A*. *toxicaria*, *A*. *ferruginea*, and *A*. *boonei*.

**Table 3:** Comparison of cocoa production systems (medium and low shade systems) along a climatic gradient from dry to mid and wet cocoa regions in Ghana (only significant differences at p<0.05 between systems are indicated by superscript letters).

Region	Dry		Mid		Wet	
Production system	Medium	Low	Medium	Low	Medium	Low Shade
Number of cocoa farms	24	21	7	42	14	34
Yield (kg ha-^1^ y^-1^)	323^c^ ±282	268 ^c^ ±265	454 ^b^ ±290	415^b^ ±275	568 ^b^ ±289	870 ^a^ ±556
Farm area (ha^-1^)	1.5±1.0	2.1±3.2	2.9±1.7	6.2±7.1	2.3±1.7	2.1±1.8
Farm age (yrs)	15±10	17±10	18±8	21±8	15±5	17±10
**Shade**						
Shade cover (%)	32.0^a^±8.1	14.0^b^±4.8	27.0^a^±8.2	11.0^b^±5.3	30.0^a^±8.4	12.0^b^±4.8
Shade tree density (ha^-1^)	57.0^a^±15.5	25.0^b^±7.1	49.0^a^±9.7	19.0^b^±8.5	58.0^a^±11.3	22.0^b^±9.6
Shade tree species (ha^-1^)	25.0^a^±12.8	14.0^b^±5.8	20.0^a^±10.8	9.0^b^±4.3	24.0^a^±7.0	12.0^b^±6.2
DBH (cm)	36.0±27.9	33.0±9.4	39.0±6.7	45.0±13.0	34.0±10.6	36.0±14.1
**Management**						
Organic fertilizer (kg ha^-1^)	0.0±0.0	0.0±0.0	20.0±55.0	45.0±91.0	40.0±80.5	35.0±135.6
Mineral fertilizer (kg ha^-1^)	5.0±19.0	0.0±0.0	0.0±0.0	25.0±78.8	45.0±83.7	45.0±114.0
Labour cost (USD ha^-1^)	41±55	47±68	56±60	29±37	24±31	43±51
Fungicide (sachets ha^-1^)	68.0±114.6	23.0±35.1	23.0±27.7	22.0±26.0	16.0±14.3	24.0±56.3
Insecticide (l ha^-1^)	6.5±14.2	2.6±2.04	3.3±2.06	2.6±1.9	3.5±2.8	13.0±56.0
**Pest and disease incidence**						
Black pod (%)	3^b^±7	5^b^±11	31^b^±18	18^b^±15	33^a^±19	17^b^±17
Capsids (%)	47±35	43±31	70±27	60±26	48±30	46±24

The low shade system had higher fertilizer input and lower labor costs (mainly associated to weeding costs) than the medium shade system with exception of the mid region where the number of farms under medium shade system were much smaller (only 7 out of 49). Fungicide and pesticide inputs were not significantly different between systems. Farm size was similar except in the wet region, where the medium shade system had a larger mean farm size. No significant differences could be observed between shade systems in the dry and mid regions with respect to black pod disease (*Phytophthora spp*.) incidence, whereas medium shade systems in the wet region displayed a significantly higher incidence of this disease. For capsids (*D*. *theobroma* and *S*. *singularis*) no significant difference could be observed between systems in all regions (Table 3).

## Discussion

The study confirms our basic hypothesis that, the climatic gradient significantly influences cocoa production, cocoa farmer income diversification and type of shade trees in cocoa agroforestry systems. Cocoa yields were low in the dry region with only half of farmers income derived from cocoa. However, farmers were adapting through diversification with sales of non-cocoa crops (such as maize, yam, cassava, plantain and vegetables) and off-farm income activities such as trading non-farm produce. In the mid and wet regions, farmers were dependent on cocoa for their incomes due to relatively higher yields under climatically suitable conditions. Perceived effects of climate change, mainly drought on cocoa production included yield decrease, increase in pests and diseases incidence, reduced cocoa pod number, weight and poor bean quality with fire as additional effect at the dry region. Farmers in the dry region kept the most shade trees on their farms, which by extension is an adaptation strategy to the marginal climatic conditions.

### Cocoa production, income diversification and perceived negative effects of climate change and drought on cocoa production

This study confirms the existence of a climate suitability gradient within the cocoa growing regions as demonstrated by Schroth et al.[4]. There were significant differences in cocoa productivity as one move from the wet to the dry regions with the wet and mid regions having high cocoa yields compared to the dry region. The high productivity levels in the wet and mid regions can be attributed to higher levels of intensification facilitated by good climatic conditions and higher institutional support through governmental and non-governmental interventions such as the Ghana Cocoa Board (COCOBOD) High Tech program, the Sustainable Tree Crops program by the World Cocoa Foundation and the Rainforest Alliance’s Certification program all directed towards productivity increases [34].

The dry region is rather marginalized due to environmental constraints that partly may have resulted to the low productivity [7]. In addition, the old age of farmers in the region constituted to a major constraint to intensive cocoa production and hence the lower yield [35]. It was observed that even though current cocoa production might not be economically viable for some farmers in the dry region, old farmers continued to “stay in cocoa” [2]. The occurrence of relatively large land sizes under newly established cocoa farms in the dry region can be attributed to the encouraging price hikes in cocoa prevailing at the time as ordinarily, farmers are incentivised by increase in cocoa prices [36] and will follow their fortunes despite projected climatic limitation [9,10]. In this region, young cocoa farms were mostly established after a short fallow and several years of food crops on land that was previously cultivated with cocoa. Farmers also established new farms occasionally on secondary forests. These secondary forests mainly developed from remnants of old cocoa farms abandoned after the 1983/84 ENSO event when most cocoa farms were destroyed by drought and wildfires in the dry region [7] and [37]. In the wet region, cocoa farm expansion has been attributed to recent deforestation [38], which is in accordance with the observed predominance of primary and secondary forests as mature cocoa farms land use history. Lack of available forested land has been identified as a major limitation to cocoa production in Ghana and Côte d’Ivoire [8]. This could also explain the reported lower number of young cocoa farms in the wet region. However, this has enhanced farmer’s act of diversification in terms of how they establish cocoa farms.

The negative effects of drought on cocoa production perceived by farmers in this study and confirmed in literature included but not limited to decreased yields, increase in the incidence of pests and diseases and wildfires [2,7]. Farmers could therefore avoid further cocoa planting if they are convinced of projected increase in drought duration and severity. Farmers’ knowledge on the influence of climate variability on cocoa production emanates from their experience with past drought events such as the 1983/4 and recently in 2015/16 [22]. Under marginal climatic conditions for cocoa production such as in the dry region, it is expected that farmers will substitute limited cocoa income with income from non-cocoa crops and off-farm activities. This was observed as an adaptation strategy in the dry region, where farmers diversified their income with 50% from cocoa and the rest from non-cocoa crops and off-farm income. Cashew (*Anacardium occidentale)*, which is known to be more drought tolerant and profitable has been identified as a potential alternative tree crop for the region[8,39].

The situation was different in the mid and wet regions, where farmers were much specialized and made approximately 80% of their annual income from cocoa farming. This could be attributed to farmers having the means of intensifying their cocoa production [39], due to high yields under optimum climatic conditions for cocoa production.

### Shade tree species functions and characterization along a climate gradient

Shade trees have been shown to perform various eco-system functions in cocoa cultivation [20]. The functions under which all trees species were categorized in this study are indication of their roles in the cocoa farm as perceived by farmers. Much shade tree usage (Table 2) especially fruit trees in the dry region could be attributed to farmers using shade trees to buffer micro climatic conditions and cocoa farm product diversification [40]. This can be referred to as plot level adaptation to marginal climatic conditions, which coincidentally has been recommended for such conditions [4,9]. Provision of ecosystem services by shade trees such as improving soil fertility with nitrogen fixing species such as *Gliricidia sepium* [20] was noted mainly in the dry region (Fig 4).

The analysis of shade trees in cocoa agroforestry systems revealed two main clusters, which were characterized as medium and low shade systems with 30% and 10% canopy cover respectively and consistent with the classification of mid-high and low shade systems identified across West Africa by Gockowski et al [41]. The medium shade system shade canopy cover is within the Rainforest Alliance and CRIG recommendations for sustainable cocoa production [42]. Farmers with medium shade cover generally reported reduced labour costs, which can be due to weed suppression and consequent reduced weeding. The fact that cocoa farm age for medium shade systems is lower than low shade systems across all regions can be partly attributed to farmers experience in changing climatic conditions [36], leading to a more positive perception on the effects of shade as adaptation strategy [40]. More medium shade system farms in the wet than the mid region can be attributed to farmers’ involvement in agroforestry interventions under Rainforest Alliance cocoa certification schemes. Such incentives encouraged farmers to maintain shade trees [34]. The Rainforest Alliance and UTZ certification projects promote shade maintenance and improvement of cocoa farm biological diversity [42]. However, the dry and mid regions represented a conventional situation of farmers’ use of shade trees as the decision of keeping shade trees were not influenced by certification. Since the climatic conditions are favourable for cocoa in the mid region, farmers did not find it necessary to maintain much shade compared to the dry region.

The two shade systems had different effects on cocoa yields. Medium shade systems had a positive effect on cocoa yields in the dry and mid regions but had a negative effect in the wet region. As a result, shade level recommendations for cocoa should therefore consider region specific climatic conditions. The main reason for shade reduction in cocoa farms is the promotion of black pod disease [43]. This was perceived by farmers as a major constraint in increasing shade levels in cocoa farms in the wet region [40]. However, different levels of shade did not influence the incidence of capsids. Therefore, it is expected that farmers will enhance shade usage as a measure of adapting their cocoa farms to climate change.

## Conclusion

Current cocoa production in Ghana is situated over a climate gradient, mostly based on annual rainfall regime. Low yields are already evident in the marginal regions of the cocoa belt in Ghana. Farmers in less climatically suitable areas like the dry regions diversify their income between cocoa, non-cocoa crops and off-farm income activities. Although the marginal areas are projected to be unsuitable for cocoa in the future, farmers continue to grow cocoa. Therefore, there is a need to bridge the information gap on projected climate suitability change awareness between farmers, researchers and other stakeholders. This could guide in developing interventions to ensure timely adaptation to climate change.

The study identified two shade systems, and reveals that medium shade systems are dominant in less climatically suitable dry region than the more suitable mid and wet region. This is an indication of farmers understanding the use of shade to adapt their cocoa farms in marginal climatic conditions. There is also a need to further explore the potential of medium shade systems as resilient and productive cocoa agroforestry system. Shade tree species that are recommended for cocoa in Ghana are not based on climatic regions, therefore limiting their use in climate change adaptation. Species specific studies on shade trees is still required to select appropriate species that will minimize potential negative effects, such as water competition and yield losses from pest and diseases under lower rainfall regimes [22]. Policies should also enhance shade tree ownership through farmers’ planting or assisted natural regeneration as an integral component of the cocoa landscapes.

## Supporting information

S1 Table Farmer interview and On-farm inventory data. (XLSX)

S1 Text Farmer interview questionnaire and on-farm data collection sheet. (PDF)
