# Supplementary material for: Characterization of cocoa production, income diversification and shade tree management along a climate gradient in Ghana
Source: PLoS One. 2018 Apr 16;13(4):e0195777. doi: 10.1371/journal.pone.0195777 (PMC5902014; doi:10.1371/journal.pone.0195777)
Supplement: S1 Text — (PDF) [file pone.0195777.s002.pdf]

PART I

1. FARMER INTERVIEW

Purpose of study explained and Verbal consent of farmers asked. Data collection then proceeds after farmer verbally agrees to participate.

1.1. General Information

Observer/Enumerator Name:______________________________ Date:____________________________

Farmer identification number:____

1.2. Location

Region: ____

District: ____

Village Name: ____

How long have you stayed? ______________________________

Cooperative / Farmer's Organisation (Name and Position): _________________________

GPS-coordinates of housing N:______________________________ E: ____________________________________

1.3. Household

Household head: __________________________ Spouse (nr. wives): ______________________________

Respondent relation to head (if not same): husband / wife / daughter / son / farm manager or care taker / ____

Gender of farmer..

Age: household head: _____________________

Migrant or Indigin ……………………………………………

Education level: household head: _____

1.4. Land holdings

Land (ha)	Total	Cultivated	Fallow	Rented/borrowed out	Other use
Owned					
Rented/borrowed in				-----------------------------	

1.5. Non-cocoa activities

1.5.1. Animal rearing

Animals	Number of animals	% contribution to household income
Goats		
Sheep		
Pigs		

Poultry (chicken, duck, etc.)		
Small animals (rabbits, guinea pigs)		
Fish (in pond)	size (m^2):	
Other:		

1.5.2. Non-cocoa crops

**Crops**	**Land area acres**	**Ownership**	**Estimated amount earn per year (GHS)**
1. Oil palm			
2. Citrus			
3. Cashew			
4. Rubber			
5. Maize			
6. Cassava			
7. Yam			
8. Plantain			
9. Vegetables			
**Intercrop (selects numbers)**			

Ownership: 1-Inherited 2-Outright purchase 3-"Abunu" sharecropping 4-Abusa caretaker 5-Other (specify

1.5.3. Other off-farm sources of income: Indicate presence and % of total revenue

**income**	**source**	**Estimated amount earn per year (GHS)**
Salary		
Pension / Retirement		
Assistance		
Small business		
Remittance		
Mining		
Others (specify)		

2.0. COCOA PRODUCTION

2.1. Schematic map of the farm (farmer should draw the relative position and distance of his plots to the housing)

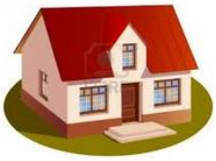

2.2. General information

Plot no. Establishment (start and end year)			Rows? Y/N & distance b/w plants	Land Tenure	Distance from household	Area acres	Type of Planting Material	Source of Planting Material	Yield (no. of bags) 2012/13	Previous land use
	Start	End								
1.										
2.										
3.										
4.										
5.										

Land tenure: 1. Own (inheritance) 2. Own (purchased) 3. Sharecropping 4. Tenancy
Previous land use: 1. Forest; 2. Long bush fallow (A minimum of 7 years); 3. Short fallow-food crop rotation; 4. Replanted old cocoa farm; 5. Abandoned cocoa farm; 6. Unknown;
Type of planting material: 1. Forastero (Amazonia); 2. Hybrid from own or neighbour (Agric); 4. Hybrid from COCOBOD seed unit;
Source of planting material: 1. Own farm or neighbours' farm; 2. Cocobod seed production unit; 3. 1 & 2 (both); 4. Unknown

2.2.1. Which months are your peak (main) and light harvesting periods? a. Peak or main harvesting months... b. light crop months..

2.2.2. Production (bags or kg) trend over the past 2years for all farms respondent not able to give yield for individual farms. 2011/12…………..2012/13…………..

2.3. Labour

2.3.1. Which household members work on the cocoa farm? (household members from youngest up)

	Gender (M/F) and Age	Level of education	Profession	Assisted with:							In total, how many days did this person work in the farm per year?*
				Weeding	pesticides & herbicide	Pruning	fertilizer	harvesting	Fermentation & Drying	transport	
1											
2											
3											
4											
5											
6											
7											
8											
9											
10											

2.3.2. Hired labour last season (2012-2013)?

(if none used check here __ and skip to next section)

	Farm activities							Total
	weeding	Pesticides & herbicide	Pruning	fertilizer	harvest	transport	Others	
# of Persons								
# of days per year?*								
Amount per day in GHS								
Cost in GHS								

2.3.3. Communal labour

	Farm activities							Total
	weeding	Pesticides & herbicide	Pruning	fertilizer	harvest	transport	Others	
# of Persons								

# of days per year?*								
Amount per day in GHS								
Cost in GHS								

2.4. Cocoa Production Training (management practices)

2.4.1. Type of cocoa farming training received (if any)...................................

2.4.2. Years of training (if applicable)...

2.4.3. Number of traininigs per year..

2.4.4. Organization ofering training..

2.5. External inputs acquired over the course of the past year (2012/13)

	Input type										
	Fertilizer-IO (Bags/kg)	Fertilizer-O (Bags/kg)	Foliar	Pesticides	herbicide	Fungicides	Equipment				
							1	2	3	4	5
Product name											
# or quantity											
Price per unit GHS											
# of application per year?*											
Period of application											
Source											

Source: 1= NGO, 2= cooperatives, 3=company, 4= local market, 5=government, 6=others – specify...
Equipment: 1=Knapsack 2=Mistblower 3=Cutlasses 4=Mistletoe remover 5=Harvesting equipment 5=Others

2.6. PLOT CHARACTERISTICS (SELECTED FARM NO......................)

2.6.1. What is the total number of cocoa trees in this farm…………………………..

2.6.2. Why did you choose this type of planting material? Availability / No other choice / Productivity / Rot Tolerance / No Reason / Other (specify) __

2.6.3. At establishment, did you plant a crop to give the cocoa trees temporary shade? YES / NO

2.6.4. If YES: what did you plant to provide temporary shade? Cassava / Plantain / Maize / Cocoyam/ Yam/ Others (specify) ____

2.6.5. After establishment, did you keep shade trees in the cocoa plot? YES / NO

2.7. EXTENSIONS

2.7.1. Have you extended the plot size since planted? YES / NO

2.7.2. If yes, have you extended the plot size: a) continuously (a bit every year) or b) discontinuously (one or more times since planting)

2.7.3. On what land use type? a. Old forest b. Fallow land/Secondary forest c. Abandoned cocoa farm d. Changed from other crop (specify)…………………..

2.7.4. What is the estimated area of the extended part?...

2.7.5. Did you use the same planting material for the extensions as at initial planting? YES / NO

2.7.6. Why did you choose this type of planting material? Availability / No other choice / Productivity / Rot Tolerance / No Reason / Other (specify)

2.8. REPLACEMENTS TO MAINTAIN/INCREASE DENSITY

2.8.1. Have you replaced cocoa trees since initial plantation to maintain/increase density of cocoa trees? _YES / NO

2.8.2. How frequently do you replace trees? Every ________ years

2.8.3. How many cocoa trees do you generally replace per year?...............................

2.8.4. Did you plant in rows? Yes No

2.8.5. If yes, at what planting distance………………………………….

2.8.6. What are you doing to increase productivity of your cocoa plot? (Tick activities)

EXTENSION	REPLACEMENT OF DEAD COCOA TREES	IMPROVE MANAGEMENT	IMPROVE INSECTICIDE TREATMENT	IMPROVE FUNGICIDE TREATMENT	FERTILISER	REDUCE SHADING	INCREASE SHADING

3. PEST AND DISEASE INCIDENCE AND MANAGEMENT

DISEASE	TIME OF YEAR	SEVERITY 1 - 5	CONTROL ACTIVITIES	PEST	TIME OF YEAR	SEVERITY 1 - 5	CONTROL ACTIVITIES
Blackpod				Capsid/Miriads			
Swollen shoot				Mealy bugs			
Dieback				Stem borers			
Leaf spot				Rodents			
				Termites			

4. FARMER ASSESSMENT OF PRODUCTION CONSTRAINTS

4.0. In the last year, which factor(s) were the most limiting to get a good cocoa production?
☐ Weather ☐ Pests & Diseases ☐ Old trees ☐ Low soil fertility ☐ Others

Annotations*____
_*Specify Climate: e.g. drought

4.1. How has the weather conditions being over the years (changes in weather conditions) 1. Fewer rains with increased temp; 2. Heavy rains and storms; 3. Unpredictable rain and dry periods; 4. Others (specify)……………………………

4.2. Since when did you start experiencing such difference?

………

4.3. Was there any negative effect on cocoa productivity on your farm caused by:

- drought event in ________(refer to most severe and recent event)? No; Yes
- the rain event in ___________ (refer to most severe and recent event)? No; Yes

If overall No → skip to next Section

4.4. If any yes to **4.3.** In general, which negative effects on cocoa production were …

… caused by drought	Severity score 1…5	…caused by excessive rain	Severity score 1…5
1 Yield decline due to lack of water		1 Yield decline due to (part of the) crop being washed away	
2 Yield decline due to more pests		2 Yield decline due to sediments covering the crop	
3 Yield decline due to more diseases		3 Yield decline due to waterlogging	
4 Yield decline due to reduced flowering/fruiting		4 Yield decline due to reduced flowering/fruiting	
5 Wild fires destroying farms		5 Soil erosion	
6 Quality (bean size)		6 Quality (bean size)	

4.5. What will you do in the case of a drought that would affect your cocoa production?

…….

4.6. If shade is not mentioned, then do you think shade trees can help reduce the effects of reduced rainfall on your cocoa? Yes No

4.7. If yes, what are your current activities on improving the shade in the farm? a. maintaining existing trees b. planting more c. Yet to start planting

4.8. Have you ever thought of changing to other crops? Yes No

4.9. If yes which crops and why?

4.10. Certification-have you heard of it?………………………………………………………………………………

4.11. Which type of certification? 1. Organic; 2. Rainforest Alliance; 3. UTZ ; 4. Others (specify)……………….

4.12. Are you certified? Which certification 1. Organic; 2. Rainforest Alliance; 3. UTZ ; 4. No certification

4.13. Have you received training on certification? When and how many times…………………………………………..

4.14. By which organization?……………………………………………………………………………………….

5. Shade trees

5.1. **Benefits and constraints**

What are the 4 main benefits of having shade trees in cocoa plots?

1………

2……….

3…….

4……..

What are the 4 main problems of having shade trees in cocoa plots?

1……..

2……..

3……..

4…….

5.2. Management of shade trees

5.2.1. Do you prune the shade trees…………………………………………………………………………………….

5.2.2. Which equipment do you use ……………………………………………………………………………

5.2.3. During which months do you do pruning…………………………………………………………………

5.2.4. What do you use the pruned materials for? ……………………………………………………

5.2.5. Which tree species do you normally prune……………………………………………………………………………………………

………

PART II

DATA COLLECTION SHEET: shade and cocoa trees

Shade and cocoa tree evaluation – on-farm **PLOT NUMBER (following the farm map): _______**

1.1. Observations on field – COCOA FARM

Location: N:______________________________ E:________________________ Altitude:____________________

1.1.1. Distance from Household to farm with GPS recording ____________________________km

1.1.2. Area of Plot (after periphery walk with GPS):____________________________________m2

1.1.3. Slope: FLAT / GENTLE SLOPE / MODERATE SLOPE / STEEP SLOPE / VERY STEEP SLOPE

1.1.4. Soil Texture: SAND / SANDY LOAM / LOAM / SANDY CLAY / CLAY LOAM / CLAY (cracking soil)

1.1.5. Erosion: NONE / SOME (rill) / MODERATE (small gully) / SEVERE (large gully)

1.1.6. Describe environment around plot: i.e. forest, fields, other cocoa plots, fallow, etc.

____

____

1.2. Shade trees

2.2.1 Inventory of shade trees in entire farm

No	Species	Canopy lengths								Dbh	Height m	Origin (0, 1)	Age
		1	2	3	4	5	6	7	8				
1.													
2.													
3.													
4.													
5.													
6.													
7.													
8.													
9.													
10.													
11.													
12.													
13.													
14.													
15.													
16.													
17.													
18.													
19.													
20.													

Origin: 0=natural 1=Planted

1.3. Cocoa trees (sampled from transect)

PROCEDURE:

1. Establish 40m baseline within the farm (avoid edges)
2. Mark five points on the line with 10m interval
3. At each point, lay two opposite lines (10m) perpendicular to the baseline
4. Record the parameters for the cocoa trees encountered on each line in the table below

Pest and disease: 0=absent 1=present

No	Dbh	Height m	Pest and Diseases						Shade tree encountered		
			Black pod (0, 1)	Severity (1 – 5)	Capisds (0, 1)	Severity (1 – 5)	Mistletoe (1, 0)	Severity (1 – 5)	Species	dbh	Dist to baseline
1.											
2.											
3.											
4.											
5.											
6.											
7.											
8.											
9.											
10.											
11.											
12.											
13.											
14.											
15.											
16.											
17.											
18.											
19.											
20.											
21.											
22.											
23.											
24.											
25.											
26.											
27.											
28.											
29.											
30.											
31.											
32.											
33.											
34.											
